# Volcanic activity sparks the Arctic Oscillation

**DOI:** 10.1038/s41598-021-94935-6

**Published:** 2021-08-04

**Authors:** Weizheng Qu, Fei Huang, Jinping Zhao, Ling Du, Yong Cao

**Affiliations:** 1 College of Oceanic and Atmospheric Sciences, Ocean University of China, Qingdao, 266100 China; 2 Physical Oceanography Laboratory/Institute of Advanced Ocean Study, Ocean University of China, Qingdao, 266100 China

**Keywords:** Climate sciences, Environmental sciences, Ocean sciences

## Abstract

The parasol effect of volcanic dust and aerosol caused by volcanic eruption results in the deepening and strengthening of the Arctic vortex system, thus stimulating or strengthening the Arctic Oscillation (AO). Three of the strongest AOs in more than a century have been linked to volcanic eruptions. Every significant fluctuation of the AO index (AOI = ΔH_middle latitudes − ΔH_Arctic) for many years has been associated with a volcanic eruption. Volcanic activity occurring at different locations in the Arctic vortex circulation will exert different effects on the polar vortex.

## Introduction

Previous research has revealed that a driving force behind the Arctic Oscillation (AO) is the self-excited behavior within the atmospheric system^1–3^. In addition, solar magnetic field anomalies and changes in magnetic field direction can also stimulate the AO to develop a corresponding 80–100-years period, and a decadal cycle consistent with the quasi-22-year cycle of the solar magnetic field direction. Because the flow of energetic particles during a solar eruption is millions of times larger than during a calm period, these particles move outward along the magnetic field lines of the Sun. When the solar magnetic field is southward, the magnetic field lines of the solar interplanetary magnetic field overlap with the magnetic field lines of the Earth, and the Earth’s magnetosphere becomes the open magnetosphere. The solar wind carries a large amount of plasma into the Earth’s magnetosphere along the magnetic field lines. Charged particles entering the earth’s magnetic field, travel along magnetic lines under the lorentz force, releasing energy between the Earth’s north and south poles and heating the atmosphere. Moreover, since the lines of force in the Earth’s magnetic field intersect the surface at the poles, high-energy particle flows not only affect the polar upper atmosphere, but are also indirect contact with the lower atmosphere, heating the polar lower atmosphere. Therefore, the polar regions are particularly sensitive to solar eruptions. That is to say, the extra solar energy input outside the Earth-air system is an important factor that stimulates the large-scale, long-period variations of the Arctic oscillation^4–6^.

Strong volcanic eruptions^7–14^, another natural phenomenon that has an important impact on the Earth’s climate, have also captured scientists’ attention. A large number of observational studies and numerical Simulation experiments have demonstrated that strong volcanic eruptions, through their stratospheric volcanic dust and aerosols, can significantly change the atmospheric radiation budget and affect large-scale and even global climate change^15–26^. Studies of the impact of volcanic activity on the North Atlantic Oscillation (NAO) have been completely indicate that, due to the middle and lower troposphere to sea level Icelandic low pressure and Azores high, winter and summer temperature and pressure field structure characteristics are basically opposite. The parasol effect of strong volcanic eruptions has led to a general drop in temperatures in the northern and southern parts of the North Atlantic, and caused opposite responses in two circulation systems, the Icelandic Low and the Azores high. For example, in winter, the Iceland low pressure system deepens as the isobaric surface of low altitude temperature drops. Azores high is a shallow system in winter. When the low air temperature drops, the high pressure active area pressurized by sea level and the high pressure system is strengthened. Thus, the reverse phase oscillation of sea level pressure field between high and low latitudes is caused, which forms or intensifies the widely influential phenomenon of NAO.

Several studies on volcanic activity and solar activity, greenhouse gas increase and digital simulation have been completed so far^27–36^, which have revealed the operational mechanism of a certain aspect of AO/NAO formation. These studies are interdecadal or interannual studies, and the general relationship between these factors and AO/NAO is revealed. This paper attempts to use synoptic methods to reveal the effects of volcanic eruptions at different locations of the atmospheric circulation system on the intensity and state of AO, which belongs to the inter-monthly scale study. It not only tells us that volcanic activity is related to AO/NAO, but also shows us how volcanic activity affects AO changes.

### Data

Volcano data compilation, Smithsonian Institution, released: Volcanoes of the World chronology. Web address is http://www.volcano.si.edu/volcanic activity and solar activity.

The global surface sea temperature data and SLP data were obtained from the Hadley Center in the UK.The website is http://hadobs.metoffice.com/hadslp2/. Other climate data were downloaded from the US National Centers for Environmental Prediction and National Center for Atmospheric Research (NCEP/NCAR).The websites: http://www.esrl.noaa.gov/psd/data/gridded/data.ncep.reanalysis.derived.pressure.html.

## Volcanic activity and the three strongest AO

**Figure 1:**
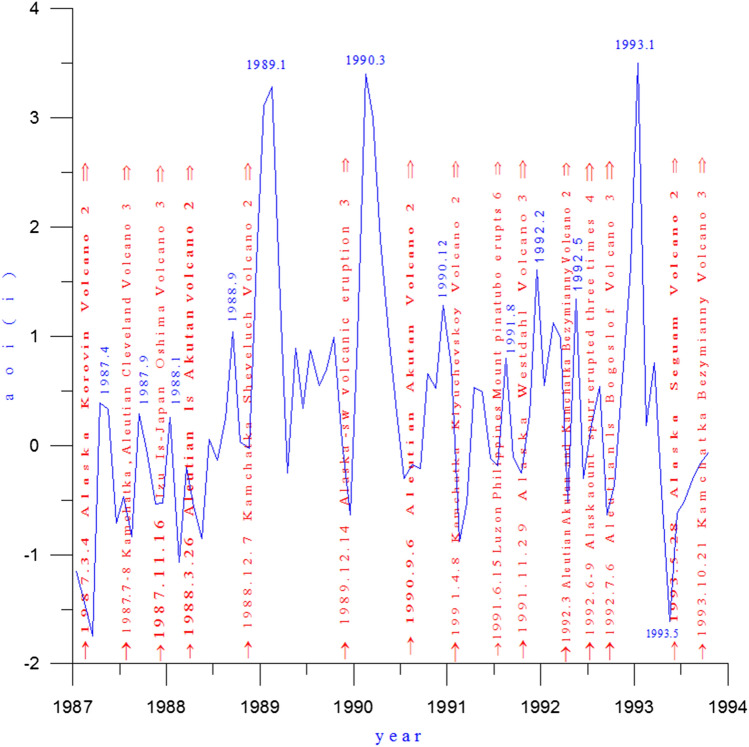
AOI (blue line) and volcanic activity (red arrows) from 1987 to 1993.

According to Table 1, the three strongest AO events since 1900, occurred in January 1993, January 1989, and March 1990, respectively. To observe the relationship between the AO and volcanic activity, the AO index (AOI) from 1987 to 1993 is presented in Fig. 1, in which volcanism of magnitude 2 or higher erupting at high latitudes in the Northern Hemisphere is plotted and marked with red arrows.

**Table 1:** The top six maximum and minimum months of the AOI from 1900 to 2015.

**AOI positive phase**							
Year.month	1993.01	1989.01	1990.03	1959.02	1957.01	2007.01	1951.12
AOI anomaly value	4.162	3.717	3.445	3.157	2.825	2.734	2.308
**AOI negative phase**							
Year.month	2010.02	1977.01	1962.03	1969.01	1966.01	2000.12	1970.03
AOI anomaly value	− 3.566	− 3.279	− 2.986	− 2.979	− 2.802	− 2.222	− 2.118

As can be seen from Fig. 1, volcanic eruptions occurred in Alaska, the Aleutian Islands, the Kamchatka Peninsula and the Kuril Islands in the northern North Pacific before the AO anomaly intensified and the AOI curve reached its maximum. The following is a detailed analysis of the role of each volcanic eruption in the anomalous intensification of the AO in combination with atmospheric circulation characteristics.

### Relationship between the strongest AO and volcanic activity in January 1993

**Figure 2:**
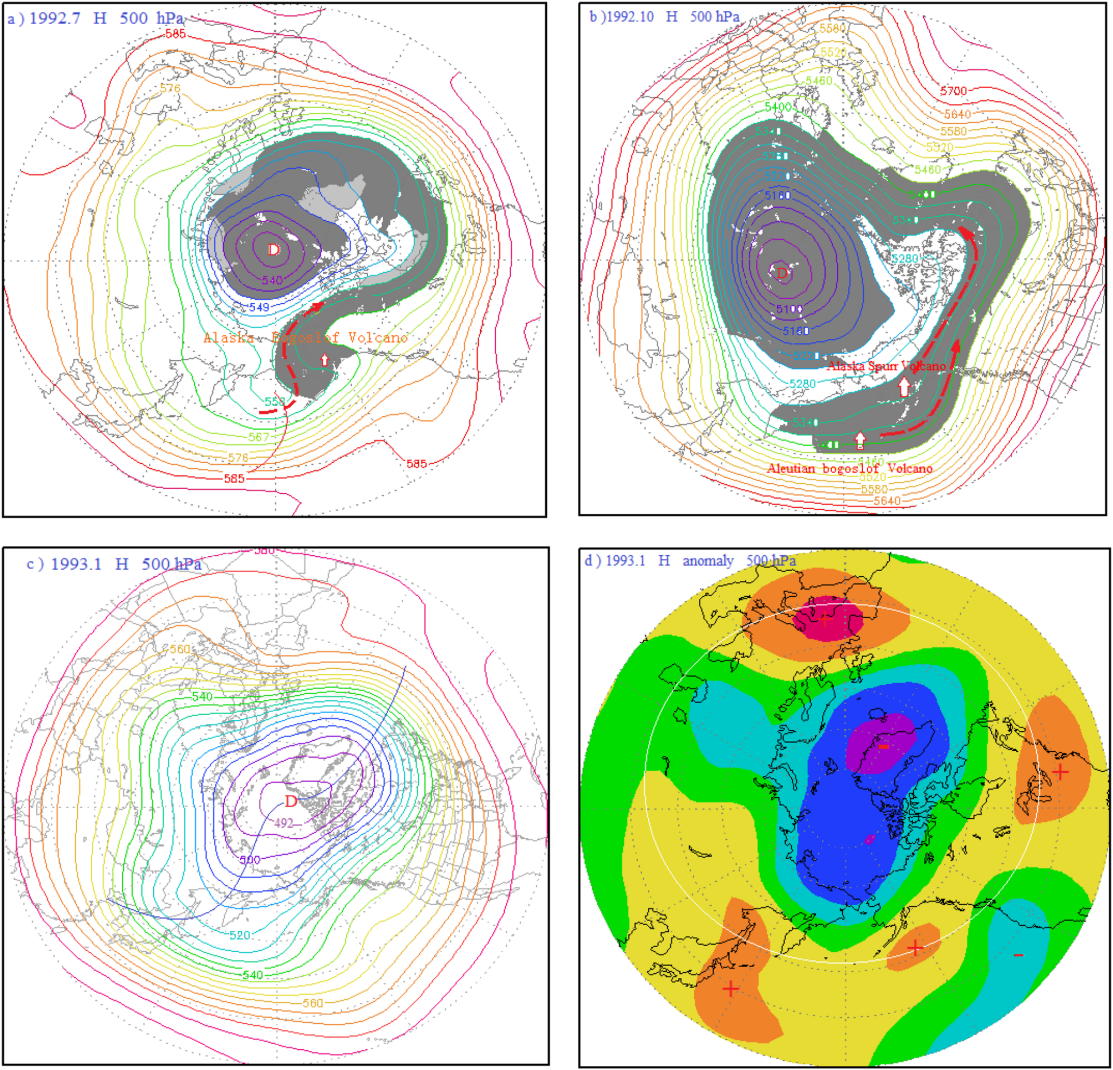
(**a**) 500 hPa atmospheric circulation characteristics and distribution diagram of the volcanic dust curtain and aerosol layer in the mid-high latitudes of the Northern Hemisphere in July 1992; (**b**) schematic diagram of the atmospheric circulation characteristics of the 500 hPa layer from 40 to 90° N and distribution of the volcanic dust curtain and aerosol layer in October 1992; (**c**) atmospheric circulation characteristics of the 500 hPa layer from 30 to 90° N in January 1993; (d) potential height field anomaly distribution of the 500 hPa layer from 30 to 90° N in January 1993.

Over the past more than a century, the AO maximum value of the AO occurred in January 1993, with the AOI anomaly of 4.162. As you can see from Fig. 1, a few months ago, it was recorded that Since 27 June 1992, Alaska Spurr volcano, located in the northern hemisphere at high latitudes, the Aleutian Bogosl of Volcano on July 6 and the Alaska Spurr Volcano on September 17 saw a series of strong eruptions. The volcanic eruption index (VEI) reaches a maximum of 4, plus the crater itself is more than 3,000 m above sea level. The volcanic dust curtain and volcanic aerosol layer into the stratosphere, reach a maximum height of more than 18,000 m. As the Alaska Volcano Spurr Volcano and the Aleutian Bogoslof Volcano take place in the polar vortex circulation (Fig. 2a,b), under the influence of the polar vortex airflow, the volcanic dust curtain and the volcanic aerosol mainly drift to the Arctic region with the airflow. In July 1992, the volcanic dust curtain and aerosol layer were drifting to the central region of the polar vortex along with the polar vortex flow, blanketing the entire Arctic region by October, as depicted by the gray dust curtain in Fig. 2a,b.

**Figure 3:**
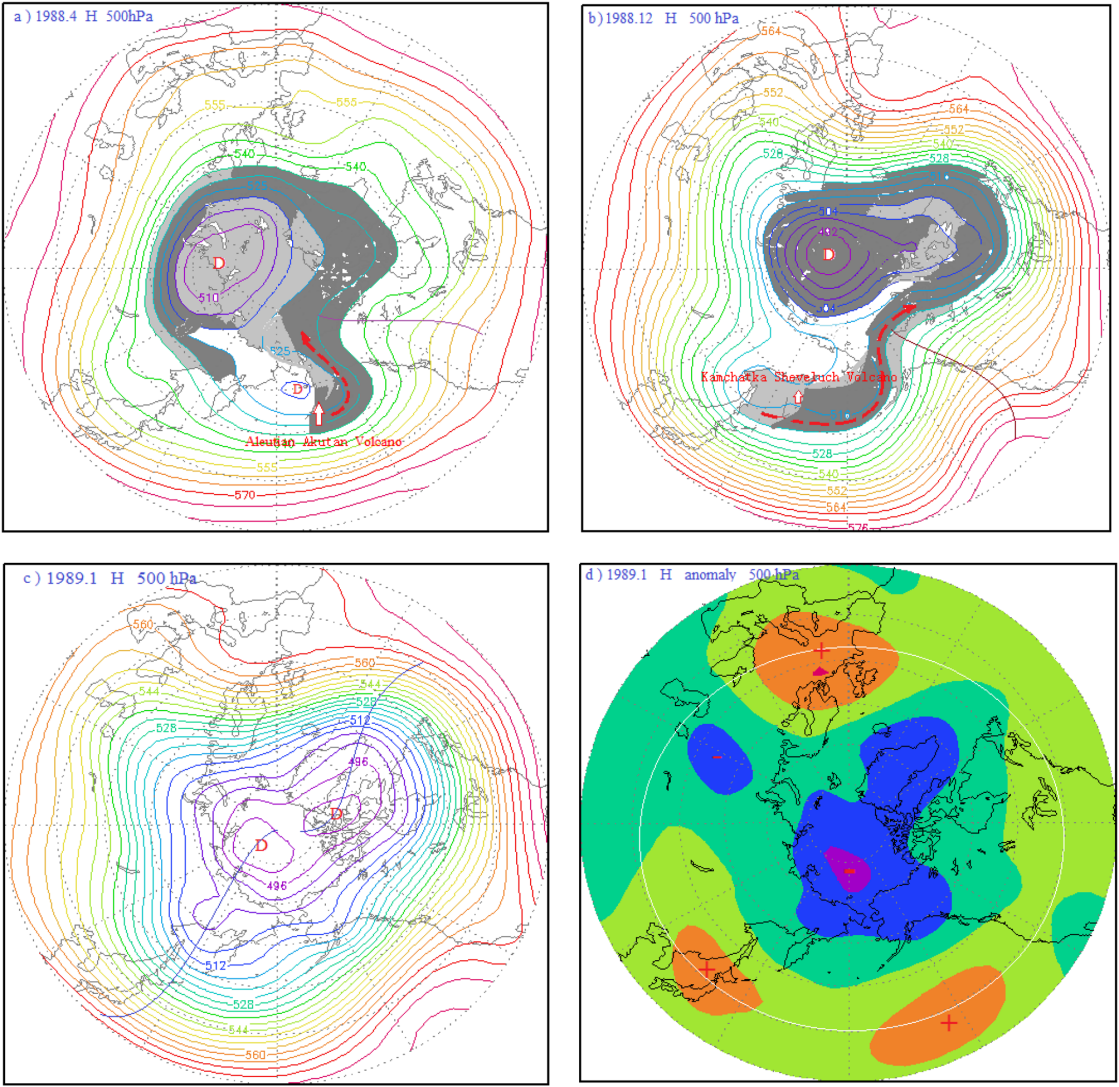
(**a**) 500 hPa atmospheric circulation characteristics and distribution diagram of the volcanic dust curtain and aerosol layer in the mid-high latitudes of the Northern Hemisphere in April 1988; (**b**) schematic diagram of the atmospheric circulation characteristics of the 500 hPa layer from 40 to 90° N and distribution of the volcanic dust curtain and aerosol layer in December 1988; (**c**) atmospheric circulation characteristics of the 500 hPa layer from 30 to 90° N in January 1989; (**d**) potential height field anomaly distribution of the 500 hPa layer from 30 to 90° N in January 1989.

**Figure 4:**
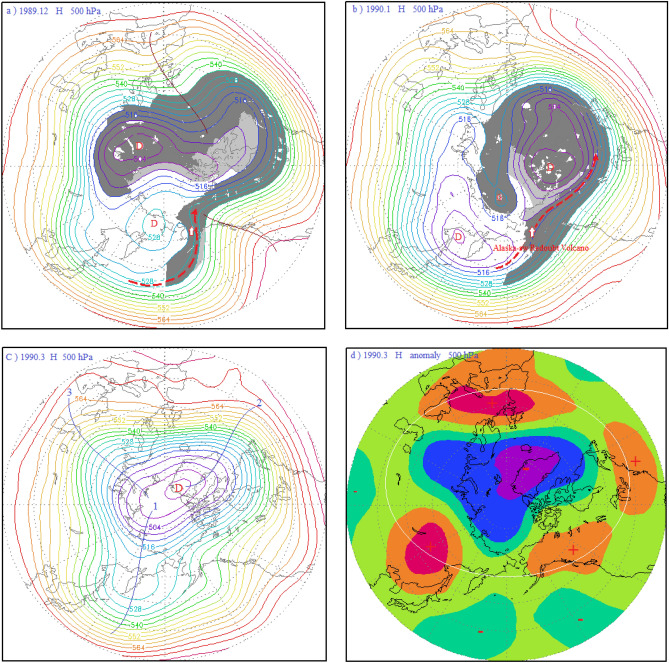
(**a**) 500 hPa atmospheric circulation characteristics and distribution diagram of the volcanic dust curtain and aerosol layer in the mid-high latitudes of the Northern Hemisphere in December 1989; (**b**) schematic diagram of the atmospheric circulation characteristics of the 500 hPa layer from 30 to 90° N and distribution of the volcanic dust curtain and aerosol layer in January 1990; (**c**) atmospheric circulation characteristics of the 500 hPa layer from 30 to 90° N in March 1990; (**d**) potential height field anomaly distribution of the 500 hPa layer from 30 to 90° N in March 1990.

**Figure 5:**
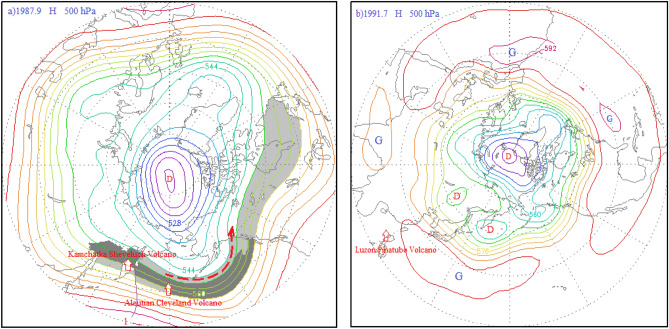
(**a**) 500 hPa atmospheric circulation characteristics and distribution diagram of the volcanic dust curtain and aerosol layer in the mid-high latitudes (40–90° N) in September 1987; (**b**) schematic diagram of the atmospheric circulation characteristics of the 500 hPa layer from 10 to 90° N in July 1991.

About topographic map drawing: The topographic maps in Figs. 2 and 3, 4, 5, 7 and 9 do not contain national boundaries and cannot be disputed because of their boundaries. Grads is a free software solution, without special authorization. GrADS Script Library: http://grads.iges.org/grads/gadoc/library.html, or http://grads.iges.org/grads.

The sun umbrella effect of volcanic dust and aerosols cools the atmosphere in the middle troposphere and the lower surface layer, resulting in the deepening of the cold polar vortex and corresponding decreases of both temperature and pressure in the Arctic region. By January 1993, the polar vortex had reached its strongest point, the mid-latitude region around the Arctic. It is a flat latitudinal circulation, as shown in Fig. 2c. The flat latitudinal circulations are not conducive to air exchange between polar cold air masses and mid-latitude warm air masses. It reduces the polar vortex pressure, strengthens the polar vortex and cools the temperature. To strengthen the high pressure in the middle latitude, temperature rise. See Fig. 2d, the green-dark blue color represents the region of negative anomaly of the pressure field, that is, the Arctic region is the region of negative anomaly; the yellow–red is the positive anomaly area of pressure field, that is, most of the mid-latitude areas around the Arctic region are the positive anomaly area of pressure field. It is shown in Fig. 2d that four positive anomaly centers of the geopotential height field in the middle latitude is arranged along the white circle, forming the positive anomaly zone of the geopotential height field around the Arctic region.

According to the AO index definition, AOI = ΔH_middle latitudes − ΔH_Arctic; thus, the higher the mid-latitude pressure, the lower the pressure in the Arctic region, and the higher the AO index. As shown in Fig. 2d, the 500 hPa geopotential height field in the mid-latitudes of the Northern Hemisphere was abnormally enhanced, while it was abnormally low in the Arctic region. As a result, the AO was unusually strong. In other words, the strong eruptions of the Alaska Spurr volcano and the Aleutian Bogoslof Volcano in the high latitudes of the Northern Hemisphere could have triggered the anomalous development of the AO.

### Relationship between the second-strongest AO and volcanic activity in January 1989

For the period extending back more than a century, the second-highest value of the AO occurred in January 1989, with an AO index of 3.717. As seen in Fig. 1, 2 months earlier, on November 16, 1987, there had been a powerful eruption of the Izu japan Oshima Volcano, with a VEI of 3. Before the volcanic dust curtain and aerosol layer had disappeared, the Aleutian Akutan Volcano erupted on March 26, 1988, erupting to a height of 5000 m, plus crater elevation of 1,300 m, volcanic dust curtain and volcanic aerosol layer reaches into the upper troposphere.

As seen in Fig. 3a, this volcanic activity occurred in the polar vortex circulation, and under the influence of the polar vortex flow, the volcanic dust and aerosols mainly spread to the Arctic region. In April 1988, the volcanic dust curtain and volcanic aerosol layer had dispersed to the central region of the polar vortex along with the polar vortex airflow. As illustrated by the gray area in Fig. 3b, following the eruption of the Kamchatka Sheveluch Volcano in December 1988, the volcanic dust and aerosol layer were further converged in the central region of the polar vortex by the polar vortex flow. The sun umbrella effect of the volcanic dust and aerosols cooled the central troposphere and lower surface layer, resulting in a deepening of the cold polar vortex in the Arctic, causing falls in temperature and pressure. By January 1989, the polar vortex was at its strongest, and there was a flat zonal flow in the mid-latitudes around the Arctic region, as shown in Fig. 3c. This flat zonal circulation was not favor to the air exchange between the polar cold air mass and the warm air mass of middle and low latitudes, and hence the polar vortex pressure was reduced and temperatures fell. Concurrently the high pressure in the mid-latitudes was strengthened, and temperatures rose. As can be seen from Fig. 3d, the height field at 500 hPa in the mid-latitudes of the Northern Hemisphere was abnormally enhanced, while being abnormally low in the Arctic region, resulting in an unusually strong Arctic Oscillation. In other words, the Aleutian Akutan Volcano and the Kamchatka Sheveluch Volcano in the high latitudes of the Northern Hemisphere have been continuously erupting, which has triggered the abnormal development of the Arctic oscillation.

### Relationship between the third-strongest AO and volcanic activity in March 1990

The third largest AO value in the past 100 years occurred in March 1990, and the AOI was 3.445. As can be seen in Fig. 4, a few months prior to this maximum, on December 7, 1989 and January 2, 1990, successive eruptions of the Alaskan Level-3 Redoubt Volcano occurred. In addition, on February 1 and March 10, 1990, the Kamchatka Kliuchevskol Volcano and the Kamchatka Level-3 Bezymianny Volcano began continuously erupting. As seen in Fig. 4a, because these volcanic activities occurred within the polar vortex, the associated volcanic dust and aerosols were spread primarily to the Arctic region by the polar vortex flow.

In December 1989, the volcanic dust and aerosol layer were spread to the central region of the polar vortex by the polar vortex airflow (Fig. 4a). In january 1990, the volcanic dust curtain and aerosol layer further converged in the central region of the polar vortex along with the polar vortex flow, as depicted by the gray dust curtain in Fig. 4b. The sun umbrella effect of the volcanic dust and aerosols cooled the atmosphere in the central troposphere and lower surface layer, resulting in the deepening of the cold polar vortex and decreasing the temperature and pressure in the Arctic region. By March 1990, the polar vortex had reached its maximum, in the form of a flat, zonal circulation extending to the mid-latitude region around the Arctic (Fig. 4c). This type of circulation is not conducive to the air exchange between the polar cold air mass and the warm air mass of middle and low latitudes. In this situation, the polar vortex region cooled, and its cyclone deepened. Concurrently, the high pressure in the mid-latitudes strengthened, and temperatures rose. As can be seen from Fig. 4d, the 500 hPa geopotential height field was abnormally enhanced in the mid-latitudes of the Northern Hemisphere, and abnormally diminished in the Arctic region. Therefore, the Arctic oscillation was unusually strong. Thus, the continuous and intense eruption of the Kamchatka Bezymianny Volcano seemingly triggered the anomalous development of the Arctic Oscillation.

Throughout this period (1987–1993), as shown in Fig. 1, each significant fluctuation of the AOI was associated with a volcanic eruption, indicating that each volcanic eruption caused an AOI increase of differing degrees.

### Varying effects of eruption location

Two volcanic eruptions during this period were not weak in intensity, but did not cause a strong AO response. Beginning on July 19, 1987, the Kamchatka Sheveluch Volcano at high latitudes in the Northern Hemisphere and the Aleutian Cleveland Volcano on August 28 produced a series of intense eruptions. Following these two volcanic events, however, the AOI only rebounded slightly. To determine why, the author mapped the 500 hPa atmospheric circulation at mid-high latitudes in the Northern Hemisphere for September 1987, as shown in Fig. 5a.

The Kamchatka Sheveluch Volcano and Aleutian Cleveland Volcano were located just on the periphery of the Arctic vortex gyre, and ahead of the ridge of high pressure, in the sinking diverging air, as depicted by ridge line 1 in Fig. 5a. As a result, only a fraction of the volcanic dust and aerosols could diffuse into the Arctic region and cause the polar vortex to deepen.

The other volcanic event was the strongest of the century, which was the eruption of the Pinatubo Volcano located in Luzon, Philippines on June 15, 1991. This eruption had a VEI of 6 and reached a height of more than 20 km, entering the upper stratosphere. This powerful volcanic eruption resulted in only small fluctuations in the AOI, however, as seen in Fig. 5b. The Luzon Philippines Pinatubo Volcano is located in a low-latitude tropical climate zone in the Northern Hemisphere. Hence, the volcanic dust curtain and aerosol layer had to pass through multiple consumption regions consisting of the tropical easterly belt, mid-latitude westerly belt, and Arctic sub-polar easterly belt in the Northern Hemisphere. Therefore, the layers of volcanic dust and aerosols that made it to the Arctic were already greatly diminished, making the effect on the polar vortex extremely small. This illustrates the great importance of volcanic eruption location.

## Volcanic activity and the two strongest negative-phase AOs

As can be seen from Table 1, the largest negative anomaly value of the AOI over the past century occurred in February 2010, when the value reached -3.566, while the second-largest negative anomaly occurred in January 1977, when it fell to -3.279. Taking the polar extreme of the anti-phase of the two AO values as an example, the role of volcanic activity in the formation of the AO negative-phase index extremum was examined.

### The formation of the anti-phase maximum of the AO in February 2010 is related to volcanic activity

**Figure 6:**
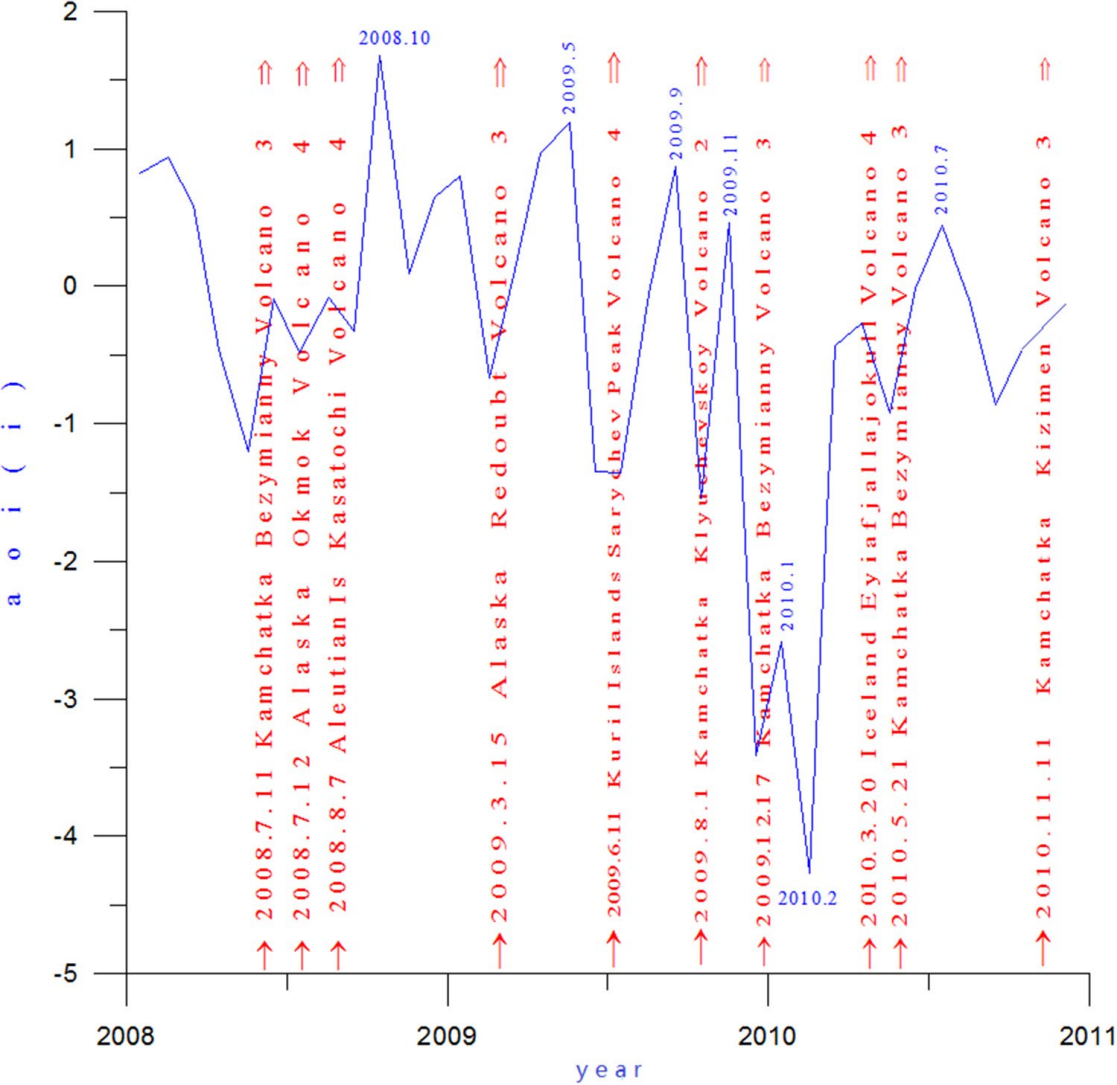
AOI (blue line) and volcanic activity (red arrows) from 2008 to 2011.

To observe the relationship between the polar oscillation minimum and volcanic activity, the volcanic activity at high latitudes in the Northern Hemisphere was added to the AOI graph for the years 2008–2011, as indicated by the red arrows in Fig. 6. The AOI maximum for this period was 1.68 in October 2008 (Fig. 6), which is the result of successive eruptions of Kamchatka Bezymianny Volcano (VEI 3) and Alaska Okmok Volcano (VEI 4) and Aleutian bogoslof Volcano (VEI 4) on July 11 solstice August 7, 2008.

**Figure 7:**
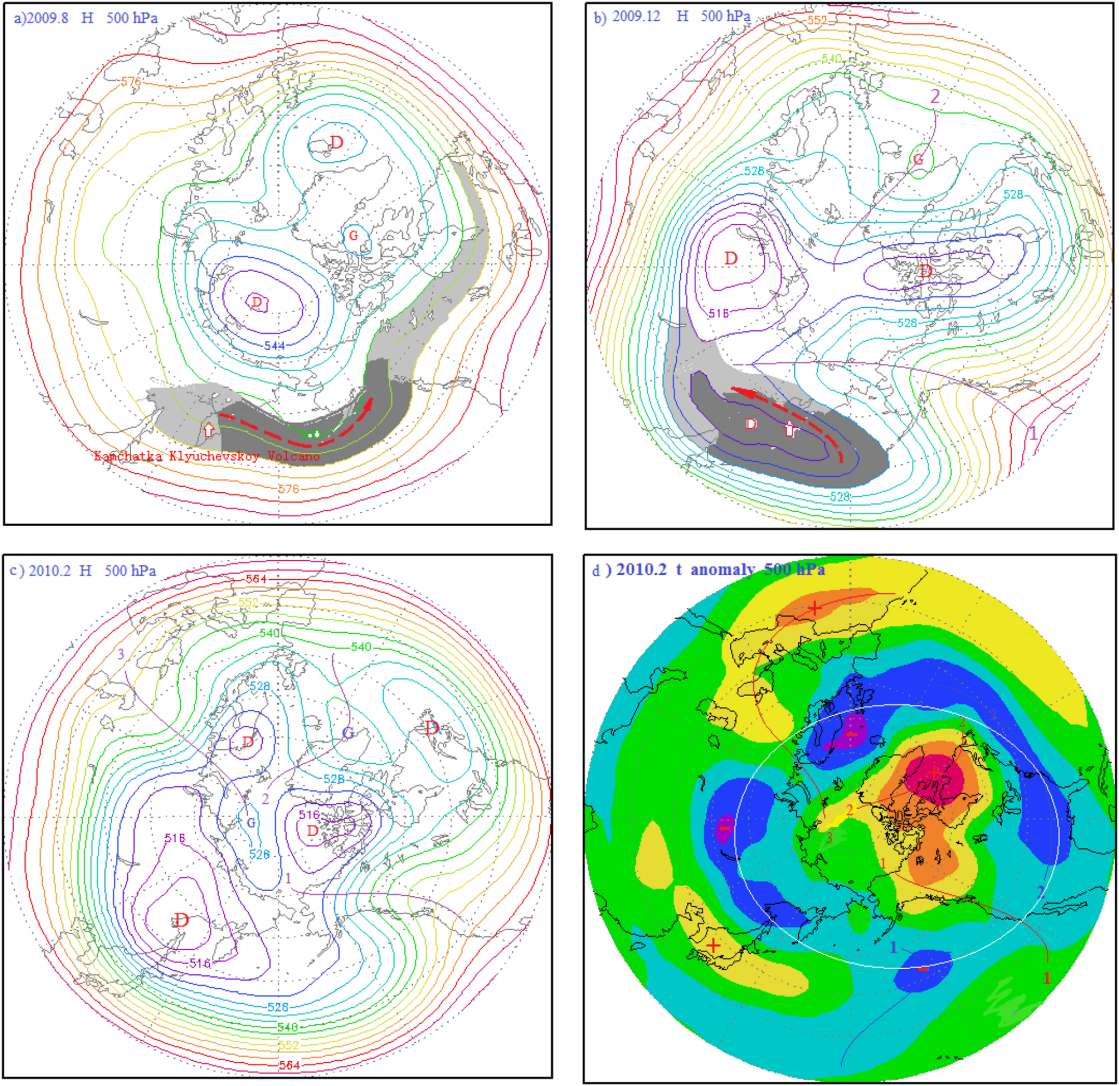
(**a**) 500 hPa atmospheric circulation characteristics and distribution diagram of the volcanic dust curtain and aerosol layer in the mid-high latitudes of the Northern Hemisphere in August 2009; (**b**) schematic diagram of the atmospheric circulation characteristics of the hPa layer from 40 to 90° N and distribution of the volcanic dust curtain and aerosol layer in December 2009; (° ) atmospheric circulation characteristics of the 500 hPa layer from 30 to 90° N in February 2010; (**d**) potential height field anomaly distribution of the 500 hPa from 30 to 90° N in February 2010.

After the AO maximum in October 2008, the AO intensity continued to fluctuate downward until February 2010, when it reached -3.566, which was the lowest AOI value since 1950. As can be seen from Fig. 6, during the continuous fluctuation of the AO intensity, each fluctuation was associated with a volcanic eruption, indicating that each volcanic eruption could cause an Arctic oscillation peak, followed by a certain amount of upswing. We will now explore this relationship in detail. The AO index continued to decline after the AO maximum in October 2008. In the mid of the falling AOI, the Alaska Redoubt Volcano (VEI 3) erupted on March 15, 2009. The volcanic dust curtain and aerosol layer then spread to the Arctic region, carried by the polar vortex airflow. In the central region of the polar vortex, the sun umbrella effect of the volcanic dust curtain and aerosol layer resulted in the deepening of the cold polar vortex in the Arctic region, decreases of atmospheric pressure and temperature, and an increase of the AOI. The AOI maximum value was 1.19 in May 2009, which was the first maximum since the AO began to decline in October 2008. After the first maximum, in the process of rapid AOI decline, Kuril Islands Sarychev Peak Volcano (VEI 4) erupted on June 11, 2009, causing AOI to rise again. A second AOI maximum value of 0.87 then occurred in September 2009. After that, the AO index resumed its decline, until another eruption of the Kamchatka Klyuchevskoy Volcano (VEI 2) occurred on August 1, 2009, causing the AOI to rebound and reach a third maximum. Because the Kamchatka Klyuchevskoy Volcano erupted in a zonal flow of air outside the polar vortex, as seen in Fig. 7a, the volcanic dust and aerosol layer were diffused along the periphery of the polar vortex by the polar vortex flow. Hence, the amount of aerosols entering the Arctic region was small. Inaddition, the eruption was not very strong, only a magnitude 2, Therefore, the upswing amplitude of the AOI curve is also very small, and the third maximum value formed is only 0.46. After this maximum, the AOI fell rapidly again. During this descent, the eruption of the Kamchatka Bezymianny Volcano (VEI 3) occurred on December 17, 2009. As a result, the volcanic dust curtain and aerosol layer only spread over the East Asian vortex. Since there was no access to the Arctic region, the resulting AOI fluctuation was small.

As seen in Fig. 7b, in December 2009, the Pacific-North American ridge of high pressure expanded Abnormally toward the Arctic region, and the polar vortex split into three vortices heading south: the North American Great Lakes vortex, the European vortex, and the East Asian vortex, with the East Asian vortex completely removed from the Arctic region. As shown in Fig. 7b, since the Kamchatka Bezymianny Volcano was located in the East Asian low eddy circulation, the volcanic dust curtain and aerosol layer could only spread within the East Asian vortex circulation and thus could not expand into the Arctic region. Moreover, the volcanic dust curtain and aerosol layer promoted further strengthening of the East Asian vortex circulation, and the southwest advection ahead of the vortex was favorable for the Pacific-North American ridge to advance toward the Arctic region, causing the isobaric surface of the Arctic region to rise. As shown in Fig. 7d, the Arctic region was controlled by the positive geopotential height field anomaly, while the middle and high latitudes around the Arctic region formed a negative geopotential height field anomaly zone. As shown in Fig. 7c, a blocking high appeared in the Arctic region, while the Great Lakes, European, and East Asian vortices surrounded the Arctic region. A strong anti-oscillation phenomenon was formed in which the atmospheric pressure in the Northern Hemisphere fell in the middle and high latitudes and rose in the Arctic region. After January 2010, the AOI declined further, reaching a minimum of -3.556 in February 2010, which was the lowest value since 1900.

From October 2008 to February 2010, the AOI curve dropped to the lowest point, forming the maximum of antiphase oscillation. During that time, a total of four eruptions occurred. Although each of these eruptions caused the AOI to temporarily rise, since their locations were farther and farther away from the center of the Arctic vortex, the amplitudes of the associated AOI fluctuations became smaller and smaller, finally reaching the minimum AOI value.

### Atmospheric circulation characteristics of the anti-phase Arctic oscillation in February 2010

Figure 7c shows the atmospheric circulation characteristics of the AO during the maximum anti-phase in February 2010. As can be seen from this figure, the meridional circulation of the mid-high latitudes in the Northern Hemisphere was developing abnormally, as was the North Pacific-North American high pressure ridge. Another ridge of high pressure in the North Atlantic also developed abnormally (Purple ridge line 2 is shown in Fig. 7c. The ridge passes through Greenland and goes deep into the arctic region, forming a closed high pressure center in the Arctic region, blocking high pressure is formed. Thus, the polar vortex was split into the Eurasian vortex and the North American Great Lakes vortex, while the Eurasian vortex moved southward, completely out of the Arctic region. The Great Lakes vortex also moved south, mostly out of the Arctic region. There was also a high pressure ridge in the Caucasus region of Eurasia moving toward the North Pole, as indicated by ridge line 3. The Eurasian vortex was split into the European vortex and the Asian vortex, and since the center of Asian vortex was in East Asia, it was also referred to as the East Asian vortex.

As a result, the arctic region is controlled by the Arctic vortex circulation during AO peak period. It gradually evolved into a period of anti oscillation under the influence of high pressure ridge or high pressure circulation. Correspondingly, the Arctic region also evolves from the negative anomaly area of potential height field in the high period of AO to the positive anomaly area of potential height field in the anti-oscillation period, see Figs. 2d, 3d, 4d, and 7d. On the contrary, the positive anomalous zone of geopotential height field around the Arctic region at middle and high latitudes during the high AO period, as shown in Figs. 2d, 3d and 4d. A positive anomaly belt composed of several positive anomaly centers connected by a white circle. It becomes the negative anomaly zone of geopotential height field in the period of anti oscillation. In Fig. 7d, the negative anomaly belt is composed of several negative anomaly centers connected by the white circle.

Throughout this period (2008–2010), as shown in Fig. 6, each significant fluctuation of the AOI was associated with a volcanic eruption, and each volcanic eruption caused AOI increases to varying degrees.

### The formation of the anti-phase maximum of the AO in January 1977 related to volcanic activity

**Figure 8:**
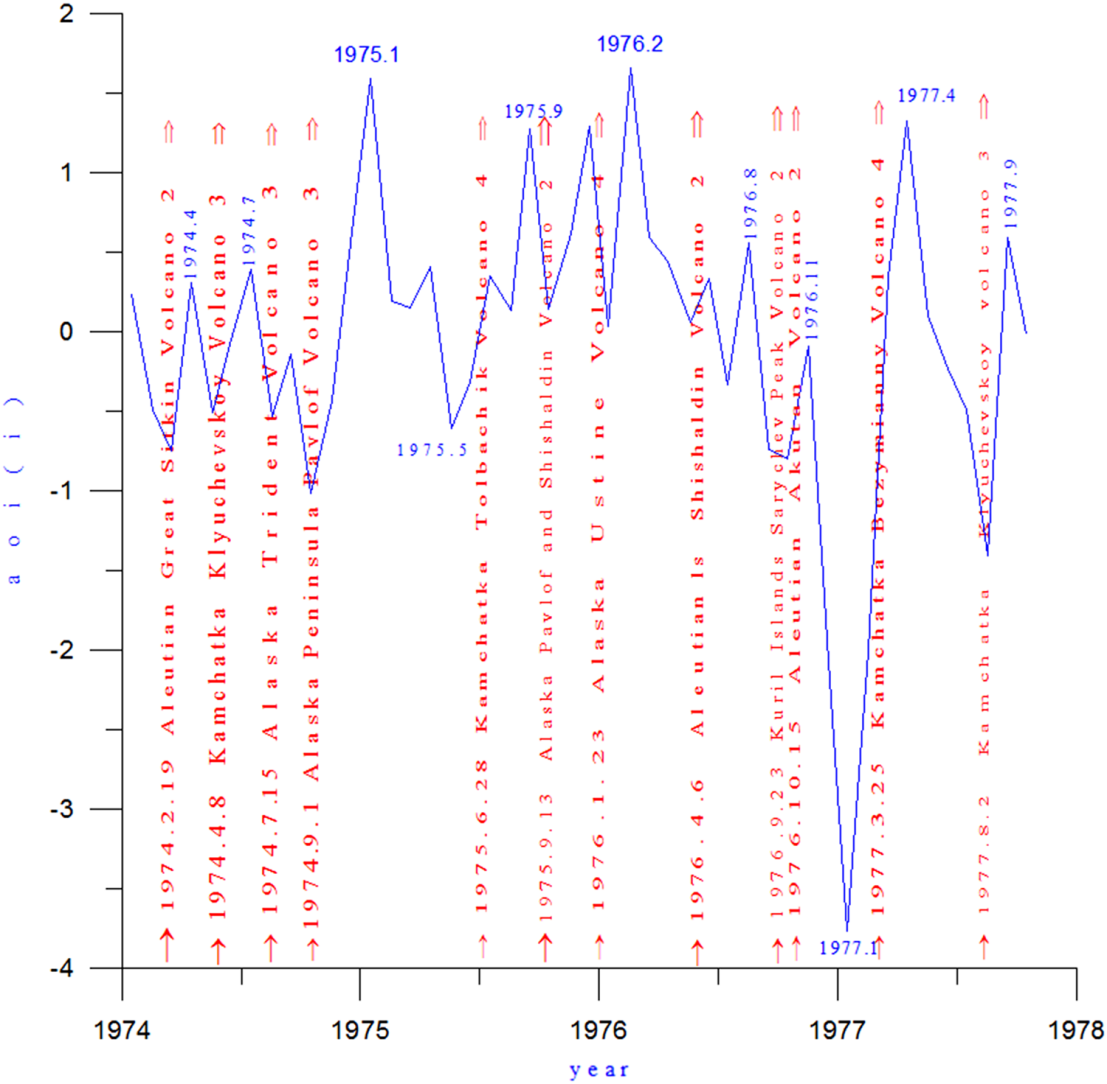
AOI (blue line) and volcanic activity (red arrows) from 1974 to 1977.

**Figure 9:**
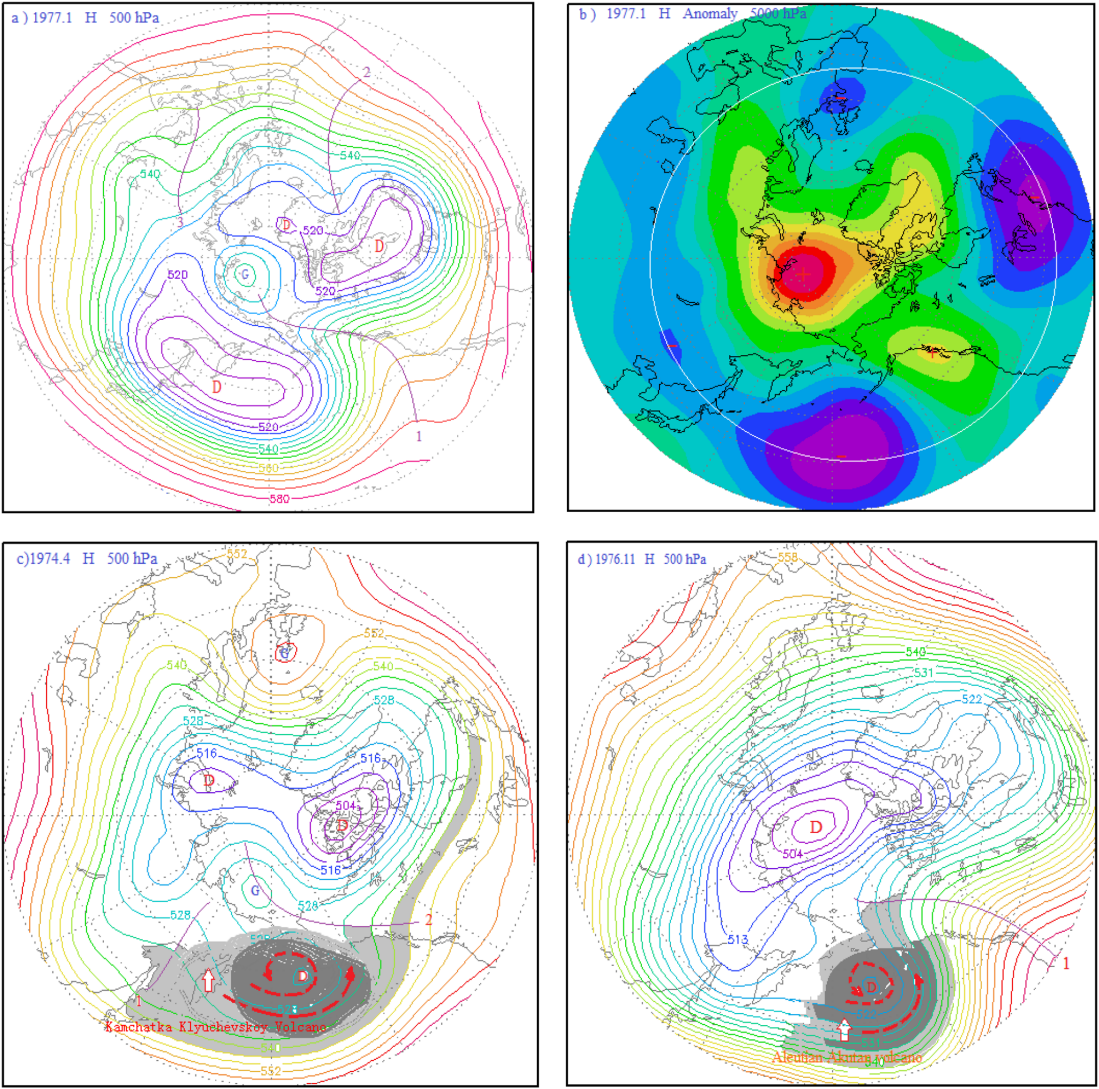
(**a**) Atmospheric circulation characteristics of the 500 hPa layer from 30 to 90° N in January 1977; (**b**) potential height field anomaly distribution of the 500 hPa layer from 30 to 90° N in January 1977; (**c**) 500 hPa atmospheric circulation characteristics and distribution diagram of volcanic dust curtain and aerosol layer in mid-high latitudes of the Northern Hemisphere in April 1974; (**d**) schematic diagram of the atmospheric circulation characteristics of the 500 hPa layer from 40 to 90° N and distribution of the volcanic dust curtain and aerosol layer in November 1976.

As seen in Fig. 8, from 1974 to 1977, the AOI maximum was 1.66, occurring in February 1976. This was related to three previous consecutive volcanic eruptions that of the Kamchatka Tolbachik Volcano (VEI 4) on June 28, 1975, the Alaska Pavlof Volcano (VEI 2) on September 13, and the Alaska Ustine Volcano (VEI 4) on January 23, 1976. After the AO maximum in February 1976, the AO intensity fluctuated continuously. By January 1977, the AOI had dropped to − 3.279, which was the second-largest AOI of the anti-phase Arctic Oscillation since 1950. As can be seen from Fig. 8, during the continuous fluctuation of the AO intensity, each fluctuation was associated with a volcanic eruption. In other words, each volcanic eruption appeared to cause an AO peak, followed by a certain amount of upswing. As an example, during the AOI decrease following the February 1976 AO maximum, the Aleutian Shishaldin Volcano (VEI 2) erupted on April 6, 1976. Because the Aleutian Shishaldin Volcano is small and erupts outside the Arctic vortex circulation, the amount of volcanic dust curtain and volcanic aerosol layer spreading to the Arctic region is small, only caused AOI index curve a small rise. By August 1976, the AOI maximum value was only 0.56, it is the first maximum since the AO began its decline in February 1976. After the first maximum, in the rapid AOI decline process, the Kuril Islands Sarychev Peak Volcano (VEI 2) erupted on 23 September 1976. Because the Kuril Islands Sarychev Peak Volcano are small in intensity, as shown in Fig. 9d, and the eruption occurred outside the Arctic vortex circulation, the amount of volcanic dust curtain and volcanic aerosol layer spreading to the Arctic region is small, AOI index curve only caused a small rise. The second AOI maximum value − 0.09 was formed in November 1976. Since then the AOI has continued to decline. In January 1977, it reached the second anti-phase maximum − 3.279 since 1900.

### Influence of volcanic eruption location on AOI

As mentioned above, the Aleutian Shishaldin Volcano erupted in the north on April 6, 1976. Since it was on the periphery of the polar vortex circulation, the size of the volcanic dust curtain and aerosol layer spreading to the Arctic region was small, causing only a small rise in the AOI. Later that year, on October 15, the Aleutian Akutan Volcano erupted, outside the Arctic vortex. As shown in Fig. 9d, a new vortex had formed off the Aleutian Islands, and the Aleutian Akutan Volcano was within the Aleutian Low eddy circulation. Hence, the volcanic dust curtain and aerosol layer could only diffuse within the Aleutian Low vortex circulation and could not enter the Arctic region. Moreover, the volcanic dust curtain and aerosol layer promoted further strengthening of the Aleutian vortex circulation, the enhanced southwest advection in front of the vortex is conducive to the further strengthening of the Pacific-North American ridge toward the Arctic region. This causes the isobaric surface of the Arctic region to rise. As shown in Fig. 9b, the Arctic region was controlled by a positive geopotential height anomaly, while a negative geopotential height anomaly zone existed in the middle and high latitudes around the Arctic region. A blocking high was present in the Arctic region, with the Great Lakes vortex and East Asian-Aleutian vortex surrounding the Arctic region. A strong anti-oscillation event was generated in which the atmospheric pressure in the Northern Hemisphere fell in the middle and high latitudes and rose in the Arctic region. In addition, the Kamchatka Klyuchevskoy Volcano erupted on April 8, 1974, although this eruption occurred outside the Arctic vortex circulation and under the subsiding diffuse flow ahead of the high pressure ridge, as shown in Fig. 9c. It is also apparent that a vortex had formed near the Aleutian Islands, and the volcanic dust and aerosol layer from the eruption circulated within the vortex. Hence, the amount of outward diffusion was small, and the effect on the center of the Arctic vortex was small.

### Atmospheric circulation characteristics of the anti-phase Arctic oscillation in January 1977

Figure 9a shows the atmospheric circulation characteristics of the Arctic Oscillation during the anti-phase period in January 1977. As can be seen from this figure, a meridional circulation anomaly at middle and high latitudes in the Northern Hemisphere had developed, and the North Pacific-North American ridge of high pressure had developed abnormally. This ridge of high pressure extended toward the Arctic region, as depicted by ridge line 1 in the figure, forming a closed high pressure center in the Arctic region, which was a blocking feature. Thus, the polar vortex was split into the Asian vortex and the North American Great Lakes vortex, with the Asian vortex pushed southward and almost entirely out of the Arctic region. Since the center of the Asian vortex is in East Asia, it is also referred to as the East Asian vortex. In addition, since the bulk of the East Asian vortex covers the Aleutian Sea area, it is also known as the Aleutian Low. The Great Lakes vortex also shifted south, mostly out of the Arctic region. Another high pressure ridge, the North Atlantic high pressure ridge, was also developing abnormally during this period, as depicted by ridge line 2 in Fig. 9a. The high pressure ridge bulged toward Greenland, making the Greenland low pressure area shallower. There was also a high pressure ridge in the Eurasian Caucasus heading toward the North Pole, as depicted by ridge 3 in the figure. Thus, the peak of the AO controlled by the Arctic vortex circulation evolved into a period of anti-phase AO influenced by a ridge or circulation. Corresponding to this, the Arctic region also evolved from the negative anomaly area of the potential height field during the peak period of the AO, as shown in Figs. 2d, 3d, and 4d, to the positive anomaly area of the potential height field during the anti-oscillation period, as shown in Fig. 9b. Contrary to this, the positive anomaly zone of the potential height field around the Arctic region at middle and high latitudes during the peak period of the AO, as shown by the positive anomaly zone composed of several positive anomaly centers connected by the white circle in Figs. 2d, 3d, and 4d, became the negative anomaly zone composed of several negative anomaly centers connected by the white circle in Fig. 9b.

Throughout this period (1974–1977), as shown in Fig. 8, each significant fluctuation of AOI curve of the Arctic Oscillation Index is associated with a volcanic eruption, or each volcanic eruption can cause a rise of AOI curve to different degrees.

## Summary

The parasol effect of volcanic aerosol layer and volcanic dust curtain caused by volcanic eruption, so that the troposphere low temperature drops, isobaric surface also drops, the Arctic vortex system deepens, To stimulate or strengthen the Arctic oscillation.

1. The strongest AO occurred in January 1993 and was triggered by the eruptions of the Alaska Spurr Volcano on June 27, 1992, the Aleutian Bogoslof Volcano on July 6, 1992, and the Alaska Spurr Volcano on September 17, 1992. The second -strongest AO occurred in January 1989 and was triggered by the continuous strong eruptions of the Aleutian Akutan Volcano on March 26, 1988 and the Kamchatka Sheveluch Volcano on December 7, 1988. And the third-strongest AO occurred in March 1990, was triggered by successive eruptions of the Alaskan Level-3 Redoubt Volcano on December 7, 1989 and January 2, 1990, and the Kamchatka Kliuchevskol Volcano Level-3 eruption on February 1, 1990. These eruptions occurred in convergent updrafts ahead of the trough and behind the ridge of high pressure in the polar vortex circulation. Under the influence of polar vortex flow, the volcanic dust and aerosols they ejected were transported to the central region of the Arctic vortex. The sun umbrella effect of these materials caused the Arctic vortex system to deepen and strengthen, thus stimulating or reinforcing the AO.
2. The longitudinal observation of each period, including the 1987–1994 period shown in Fig. 1, the 2008–2011 period in Fig. 6, and the 1974–1978 period in Fig. 8, revealed that every significant AOI fluctuation was associated with a volcanic eruption. In other words, each volcanic eruption apparently caused an AOI increase of varying amounts.
3. Influence of volcanic eruption location on the AOI:
  - If a volcanic eruption occurs in convergent updrafts ahead of low pressure troughs and behind high pressure ridges in the Arctic vortex circulation, the volcanic dust and aerosols converge and diffuse rapidly toward the central Arctic vortex, covering its central region of the Arctic vortex, as shown in Figs. 2a,b, 3a,b, and 4a,b. The sun umbrella effect of these materials causes the Arctic vortex system to deepen and strengthen, thus stimulating or reinforcing the Arctic Oscillation phenomenon.
  - If a volcanic eruption occurs on the periphery of the Arctic vortex circulation, the volcanic dust curtain and aerosol layer are mostly dispersed and disappear along the periphery of the Arctic vortex circulation. As seen in Figs. 5a and 7a, since only a small portion of these materials enter the vortex center, this volcanic activity has little impact and causes only small AOI fluctuations.
  - If a volcanic eruption occurs in the downward-dispersive flow ahead of the ridge of the Arctic vortex circulation, most of the volcanic dust and aerosols will disappear in the downward-dispersive flow, as shown in Figs. 5a, 7a, and 9a, and only a small portion will enter the vortex center, causing only small AOI fluctuations.
  - An abnormal development of the Pacific-North American ridge of high pressure deep into the Arctic region can split the original polar vortex into two or three sections. The split polar vortex moves south, away from the North Pole and generally away from the Arctic region, where it becomes the North American Great Lakes vortex or the East Asian vortex. If a volcanic eruption occurs during this type of vortex circulation pattern, e.g., the Aleutian Akutan Volcano eruption on October 15, 1976 shown in Fig. 9d or the Kamchatka Bezymianny Volcano eruption on December 17, 2009 shown in Fig. 7b, and it is within the East Asian vortex circulation, the volcanic dust curtain and aerosol layer can only diffuse within the East Asian vortex circulation and cannot enter the Arctic region. Moreover, the volcanic dust curtain and aerosol layer will promote further strengthening of the East Asian vortex circulation, and the enhanced southwest advection ahead of the vortex will be conducive to further strengthening of the Pacific-North American ridge, expanding it toward the Arctic region. As a result, the Arctic region will become completely controlled by the high pressure circulation, generating the anti-oscillation phenomenon.

## Data Availability

The sea ice and SLP data is sourced from the Hadley Centre^5^: http://hadobs.metoffice.com/hadslp2/. Other marine climatic data is obtained from National Centers for Environmental Prediction (NCEP/) and National Center for Atmospheric Research (NCAR). These websites are: http://iridl.ldeo.columbia.edu/SOURCES/.UMD/.Carton/.goa/http://www.esrl.noaa.gov/psd/data/gridded/data.ncep.reanalysis.derived.pressure.html/.
